# Relationship between circulating microparticles and hypertension and other cardiac disease biomarkers in the elderly

**DOI:** 10.1186/s12872-019-1148-6

**Published:** 2019-07-09

**Authors:** Hanife Usta Atmaca, Feray Akbas, Hale Aral

**Affiliations:** 1 Istanbul Training and Research Hospital Internal Medicine Department, Health Sciences University, Samatya, Istanbul, Turkey; 2 Istanbul Training and Research Hospital Biochemistry Department, Health Sciences University, Istanbul, Turkey

**Keywords:** Microparticles, Endothelial dysfunction, Hypertension in the elderly, Thrombin generation test, Procoagulant activity

## Abstract

**Background:**

Microparticles are procoagulant membrane vesicles that play role in endothelium dysfunction pathogenesis and are increased in hypertension, acute/chronic vascular pathological events. Here; we aimed to compare MPs levels of hypertensive geriatric patients with healthy age-match-patients, discuss its availability as a cardiovascular biomarker and investigate its relationship with other inflammatory markers.

**Methods:**

Forty seven hypertensive geriatric patients (M/F;15/32) and 47 healthy controls (M/F;19/28) were included in the study. MPs levels were examined functionally through thrombin generation test (TGT) parameters (MPS Lag time, MPS ETP, MPs Peak, MPS start Tail) and compared with CRP, N/L ratio, ALT, GGT, thrombocyte parameters. Decrease in MPS Lag time, increase in MPS ETS and MPs Peak elevation were accepted as tendency to coagulation which meant an increase in number and function of MPs.

**Results:**

No significant difference was found between 2 groups for MPS tests (MPS Lag time, MPS ETP, MPs Peak, MPS start Tail). Platelet count was significantly higher in hypertensive patient group. There was a negative correlation between age and MPs Peak, MPS Lag time. There was a positive correlation between CRP and MPS ETP, MPs Peak values.

**Conclusions:**

Our present findings might help to understand the hemostasis via TGT parameters, in the elderly. Contribution of MPs to thrombosis tendency seen with aging and increased number of circulating MPs caused by hypertensive endothelial dysfunction must be taken into consideration. MPs might be accepted as vascular inflammation and damage markers and used as follow up tools of medical treatment of vascular inflammation-related diseases.

## Background

Microparticles (MPs) which are also called microvesicles are procoagulant membrane vesicles that are directly secreted from cell membrane by exocytic budding when apoptosis and cellular activation occur [1, 2]. They don’t have a nucleus but other cytoplasmic parts and their size is 0.1–1.0 μm [3]. They are secreted from thrombocyte, leukocyte and endothelium. They play roles in endothelium dysfunction pathogenesis and are increased in hypertension, acute and chronic vascular pathological events and hypercoagulation. They have procoagulant activity via tissue factor (TF) they carry on their surface and the negative procoagulant phospholipids (PPL) like phosphatidylserine. They are also increased in different pathophysiologic events (inflammation, coagulation and metastatic cancers) [4] and play roles in diabetic complications [5, 6].

Aging causes changes in thrombocyte function, increase in coagulation proteins and fibrinolysis dysfunction which result in a change in vascular, hemostatic and coagulation system [7–9]. Thus; arterial and venous thrombotic events are increased, endothelial thickness is increased [10] and endothelium related relaxation is decreased [11, 12]. Although increased procoagulant activity in aging is known, there is not sufficient data about the contribution of change in MPs activity to coagulation. It is shown that endothelial derived MPs (EMPs) are decreased but MPs procoagulant activity persisted in aging [13].

Hypertension is characterized with early endothelial dysfunction and is a strong risk factor for atherosclerosis, vascular morbidity and mortality. Nitric oxide (NO) is decreased in vascular wall and free O2 radicals are increased and protective effect of vascular wall is diminished. [14, 15] MPs are secreted to circulation from activated endothelium and it is suggested that they could be an early biomarker for endothelial dysfunction. Increase in the number of MPs is shown to be related with endothelial dysfunction like atherosclerosis and pulmonary hypertension [16, 17].

The assessment of thrombin generation test (TGT) is currently regarded as a useful tool for screening, diagnosis and therapeutic monitoring of a variety of hemostatic disorders; it is believed to reflect more closely the impairment between procoagulant and anticoagulant forces in vivo. Thrombin generation also seems more sensitive to fluctuations of clotting function in a major area of clinical interest that is the population of the subjects with normal values for routine clotting tests [18].

Here; it is aimed to compare MPs levels of hypertensive geriatric patients with healthy age match patients, to discuss its availability as a cardiovascular biomarker and investigate its relationship with other inflammatory markers.

## Methods

Study population:

This is a randomized, cross-sectional, case-control study. Ethical committee of Istanbul Training and Research Hospital reviewed and approved the study. All patients gave written informed consent to take part in this study. The study was conducted in accordance with 1964 Helsinki Declaration.

Hypertensive patients (over 65 years) with no any additional chronic disease, who were seen with different reasons in internal medicine outpatient clinic were included the study randomly. Forty-seven patients who had monotherapy (ace inhibitor, arb blockers, calcium channel blockers) or combined treatment (2 or more), and 47 healthy controls were enrolled. Inclusion criteria were: At least 15-years of hypertension history, geriatric patients, blood pressure within normal range under treatment. Exclusion criteria: Smoking history, alcohol consumption history, present pregnancy, accompanying any other chronic diseases (e.g. cardiac diseases, COPD, malignancy, uremia), uncontrolled hypertension, newly diagnosed hypertension, usage of drugs that would affect coagulation.

### Blood tests

Peripheral venous blood samples were drawn from all subjects after 12 h of hunger at sitting position from antecubital vein and were collected in trisodium citrate-containing tubes (0.109 M). They were centrifuged immediately after blood collection. The platelet poor plasma (PPP) was prepared by double centrifugation at 2,500 g (15 min), and the upper 2/3 supernatant was stored at − 80 °C within 2 h of collection (for less than 5 months’ time). Frozen samples were thawed in a 37 °C water bath for 5 min and vortexed before the study.

Routine biochemistry and blood count parameters were performed via AU 2700 (Beckman Coulter Inc.) and Sysmex XE 5000 (Sysmex Medical Int.). MPs levels were examined functionally through TGT parameters (Diagnostica Stago).

**Fig. 1:**
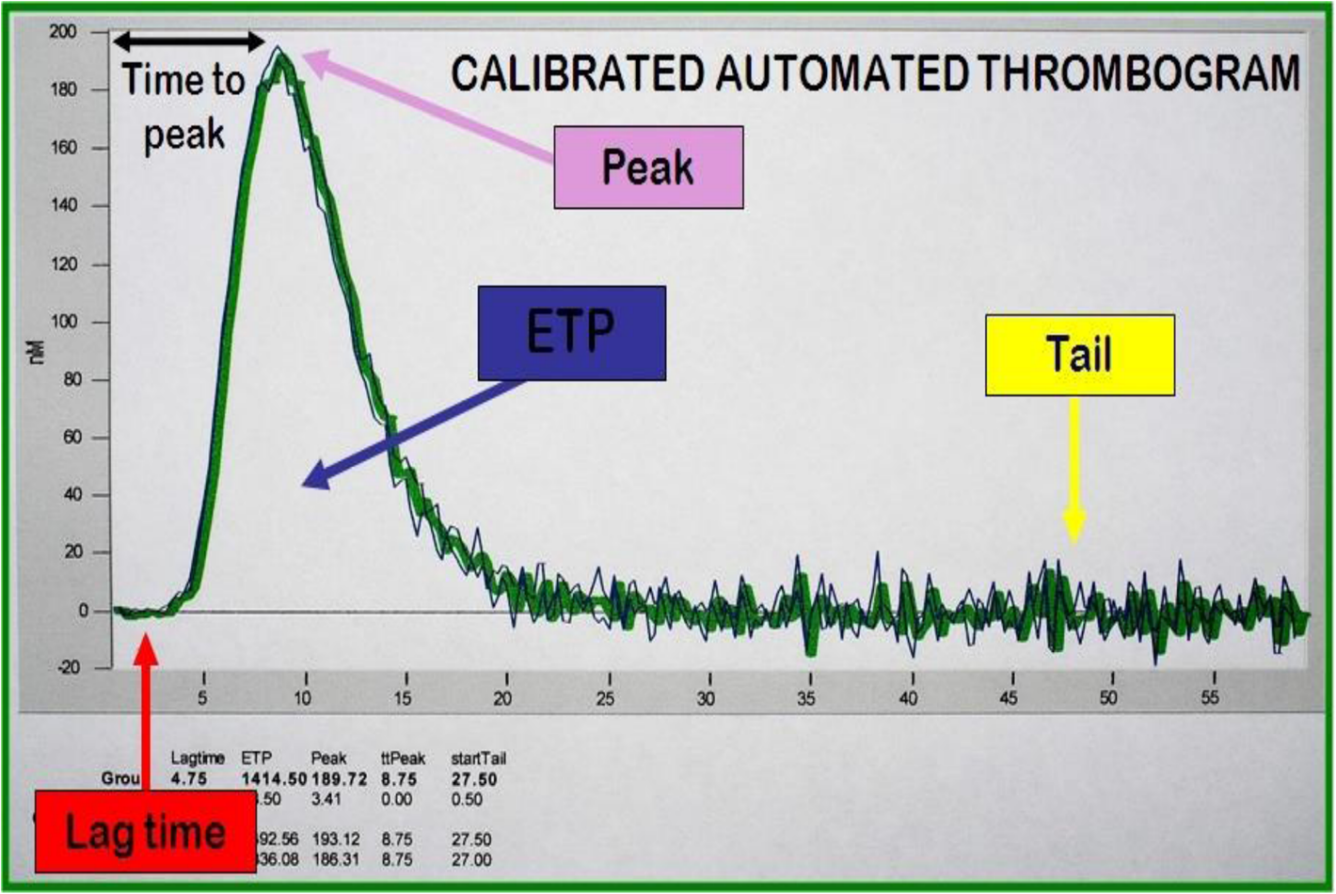
‘Thrombogram curve’ sample from CAT (Calibrated Automated Thrombography device, Diagnostica Stago, France)

Samples were dissolved in room temperature and Tissue Factor (TF) activity flourogenic measurement of TGT -developed by Dr. Hemker- was studied functionally via Calibrated Automated Thrombography (CAT, Diagnostica Stago). Following thrombogram parameters were formed according to thrombin generation curve (Fig. 1).

Calibrated Automated Thrombography (CAT, Diagnostica Stago) method that was developed by Dr. Hemker was used for Tissue Factor (TF) activity flourogenic measurement of TGT after dissolving the samples in room temperature. The CAT method enables the quantification of thrombin concentrations in platelet-rich (PRP) or platelet-poor plasma (PPP). This thrombin calibrator contains a thrombin-like enzyme linked to alpha-2-macroglobulin that isn’t inhibited by plasma components and reacts only with the fluorogenic substrate. The addition of tissue factor (TF), phospholipids (amplify the effect of TF) and calcium in the plasma, results in coagulation activation and subsequent generation of thrombin. Thrombin cleaves the fluorescent substrate (Z-Gly-Gly-Arg 7-amino-4-methylcoumarin) that is added to the reaction in a later step, releasing a fluorophore whose fluorescence intensity over time is proportional to the concentration of thrombin formed [19].

In each case sample, we used a calibrator for correction of substrate consumption, optical artifacts (plasma color, intrinsic-filter effects, etc.). So, two wells were spent for a case; the calibrator was manually pipetted to one well and MPs kit was to the next well and 80 μL of PPP samples were pipetted into the two wells. Thus, a plate (96 wells) was used for the PPP sample of 48 cases. But the thrombogram was frequently repeated, approximately in a proportion of 35%, when there was no reaction.

- Lag time (minutes): the time passed till the reaction in device starts; 1/6 (16.7%) of peak time (in x-axis)
- Endogenous Thrombin Potential (ETP) (nmol/L·minutes): area under thrombogram curve
- Peak height (nmol/L): peak height of thrombogram curve (in y-axis)
- Time to thrombogram Peak (ttPeak) (minutes): time to reach to thrombogram curve peak point (in x-axis)
- StartTail (minutes): the time in thrombogram curve is completed (in x-axis).

### Statistical analysis

Statistical analysis was performed by using the program MedCalc (MedCalc Software, Broekstraat, Mariakerke, Belgium). Values with gaussian distribution were shown as mean ± SD, and values with non-gaussian distribution were shown as median (25th percentile –75th percentile). Student’s T test, Mann-Whitney U test and chi-square test were used in comparison of the groups. Correlation was examined via Spearman correlation coefficient (rs) or Pearson correlation coefficient (r). Statistical meaningful was evaluated at *p* < 0.05 (two-tailed).

## Results

The demographic and laboratory parameters studied in the research were not statistically different between patient and control groups except for PLT (Table 1). The only correlation between PLT count and MPs Peak was in the control group (r_s_ = 0.324 *p* = 0.026). Correlations between PLT indices of PDW, MPV and the TGT parameters seemed better in the control group; as shown in Table 2 with MPs ETP (r_s_ = − 0.401 *p* = 0.006; r = − 0.364 *p* = 0.013, respectively), and with MPs Peak (r_s_ = − 0.445 *p* = 0.002; r_s_ = − 0.478 *p* < 0.001, respectively).

**Table 1:** Comparison of the demographic and laboratory data in control and patient groups

	Control group (*N* = 47)	Patient group (*N* = 47)	
Age (years)	71.6 ± 9.1	72.7 ± 7.2	= 0.5160
Gender (M/F)	19/28	15/32	= 0.3910
ALT (U/L)	18 (14–22)	16 (13–21)	= 0.3040
GGT (U/L)	20 (15–35)	19 (15–32)	= 0.6270
CRP (mg/L)	4.09 (1.83–7.76)	5.20 (2.39–10.80)	= 0.3100
WBC (×10^9/L)	7.2 ± 2.0	7.4 ± 2.0	= 0.6120
Neutrophyl (×10^9/L)	4.3 ± 1.8	4.5 ± 1.8	= 0.5880
Lymphocyte (×10^9/L)	2.1 ± 0.7	2.1 ± 0.8	= 0.9360
NLR	2.07 (1.34–2.81)	2.13 (1.41–3.09)	= 0.5010
PLT (×10^9/L)	216.9 ± 57.4	247.2 ± 64.7	**= 0.0250**
PDW (fL)	11.5 (11.0–13.3)	11.7 (10.9–13.3)	= 0.8050
MPV (fL)	10.5 ± 0.9	10.3 ± 0.8	= 0.4810
PCT (%)	0.23 (0.21–0.25)	0.26 (0.20–0.28)	= 0.1650
MPslag time (min)	12.87 (10.33–15.67)	12.00 (10.67–14.00)	= 0.5960
MPs ETP (nmol/L•min)	2092.8 ± 676.1	2029.5 ± 615.9	= 0.6450
MPsPeak (nmol/L)	377.43 (294.73–463.98)	388.27 (315.29–473.32)	= 0.5040
MPsttPeak (min)	15.67 (13.00–19.21)	14.44 (13.00–16.38)	= 0.2700
MPsstartTail (min)	31 (28–35)	30 (27–33)	= 0.2730

Meaningful difference was found for only PLT between patient and control groups

**Table 2:** Meaningful correlations found between TGT parameters of MPslag time, ETP, Peak, ttPeak, startTail parameters and other demographic/laboratory data

Variables		Whole group	Control group	Patient group
MPs lag time	Age	r_s_ = − 0.217 *p* = 0.038	r_s_ = − 0.273 *p* = 0.063	
	Lymphocyte			r = − 0.377 *p* = 0.015
MPs ETP	CRP	r_s_ = 0.393 *p* < 0.0001	r_s_ = 0.472 *p* < 0.001	
	PDW	r_s_ = − 0.273 *p* = 0.015	r_s_ = − 0.401 *p* = 0.006	
	MPV	r_s_ = − 0.271 *p* = 0.015	r = − 0.364 *p* = 0.013	
	PCT			r = − 0.374 p = 0.029
MPs Peak	CRP	r_s_ = 0.308 *p* = 0.004	r_s_ = 0.332 *p* = 0.023	
	PLT		r_s_ = 0.324 *p* = 0.026	
	PDW	r_s_ = − 0.233 *p* = 0.035	r_s_ = − 0.445 *p* = 0.002	
	MPV	r_s_ = − 0.272 *p* = 0.013	r_s_ = − 0.478 *p* < 0.001	
MPs ttPeak	Age	r_s_ = − 0.230 *p* = 0.028		
MPs startTail	Age	r_s_ = −0.261 *p* = 0.013	r_s_ = − 0.350 *p* = 0.016	
	CRP	r_s_ = 0.238 *p* = 0.028	r_s_ = 0.314 *p* = 0.032	

*r* Pearson correlation coefficient

*r*_*s*_ Spearman correlation coefficient

Although weak and negative correlations were found between age and three MPs parameters of lag time, ttPeak, startTail in the whole group (r_s_ = − 0.217 *p* = 0.038; r_s_ = − 0.230 *p* = 0.028; r_s_ = − 0.261 *p* = 0.013, respectively), they disappeared in the patient group, as shown in Table 2. mild and negative correlations were. Also, positive correlations between CRP and three MPs parameters of ETP, Peak, startTail in the whole group (r_s_ = 0.393 *p* < 0.0001; r_s_ = 0.308 *p* = 0.004; r_s_ = 0.238 *p* = 0.028, respectively) disappeared in the patient group.

In the patient group, the two meaningful correlations were weak and negative between MPs lag time and lymphocyte count (r = − 0.377 *p* = 0.015), and between ETP and PCT (r = − 0.374 *p* = 0.029).

**Fig. 2:**
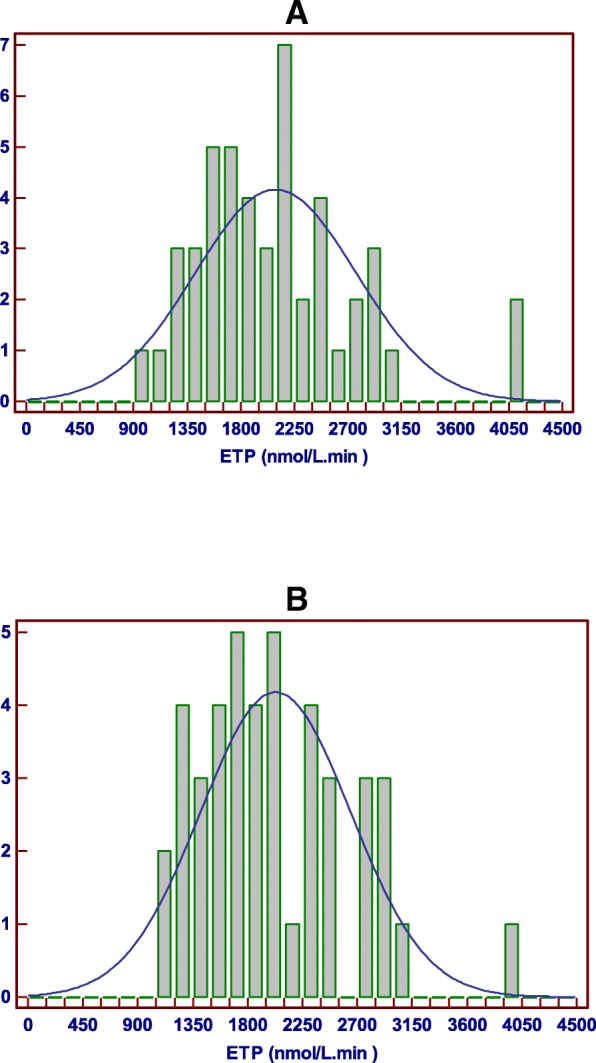
Histograms of theMPs ETP (nmol/L•dk) in the control (**a**) and the patient (**b**) groups. Skewness_Control Group_ = 1.067 Skewness_PatientGroup_ = 0.819. Kurtosis_Control Group_ = 1.775 Kurtosis_PatientGroup_ = 0.628

Examining whether the MPs parameters showed the gaussian distribution using Colmagorov-simirnov test, only the ETP presented gaussian distribution. On the other hand, when we examined histograms of both patients and controls separately, kurtosis of the MPs ETP was found more acceptable in the controls (being closer to zero) as shown in Fig. 2.

We noticed that kurtosis of the patient group seemed higher only in lag time (Fig. 1); on the contrary, kurtosis of the ETP (Fig. 2) seemed relatively lower in comparison with the controls.

## Discussion

The assessment of thrombin generation is currently regarded as a useful tool for screening, diagnosis, and therapeutic monitoring of a variety of hemostatic disorders, both hemorrhagic and prothrombotic [20] and also thrombin generation is believed to more closely reflect an impairment between procoagulant and anticoagulant forces in vivo [21]. Thrombogram obtained with this method provides several parameters. Time until thrombin is generated (lag time), thrombin generation peak time (peak time) and the area under the thrombin generation curve, which is conventionally referred to as ETP main parameters [21]. Decrease in the lag time, increase in ETP area, higher Peak values are considered as favorable to hypercoagulability, while increased lag time, decreased ETP area or Peak values reflect hypocoagulability (prohaemorrhagic state) [22]. In this study, via thrombin generation test, we have found that there is no significant difference in lag time, peak time, and etp values in old hypertensive patient and healthy subject group. On the other hand, it has been noticed that there is a high thrombocyte count found in hypertensive patient group. In the correlation analysis, there were negative correlation with age and peak & lag time in all the groups.

As the endothelial structure changes, people are more prone to coagulation within aging. Thus, a change in the number and functional state of MPs is expected. Endothelial dysfunction seen in hypertension affects MPs levels and increases risk of thrombosis. MPs are known to increase in the diseases with vascular origin like hypertension, atherosclerosis and coronary artery disease [16, 17, 23].

Previous studies have demonstrated an impairment of cell proliferation, migration, tube formation, and sprouting in older individuals (> 65 years, male or female) when compared to their younger counterparts (< 65 years, male or female) suggesting that these changes contribute to the decrease in effective blood vessel growth and repair mechanisms in the elderly [24]. It is shown that proangiogenic factors are decreased and circulating endothelial progenitor cells (EPCs) are vanished [25]. Age-related changes in EPCs number and function may directly correlate with the degree of senescent endothelial impairment [26]. MPs also play a role in angiogenesis impairment with aging. MPs can act on angiogenesis directly through ligand/receptor interaction or indirectly by modulating production of soluble factors involved in endothelial cell differentiation, proliferation, migration, and adhesion [27]. Defective angiogenesis exist in some vascular diseases and endothelial MPs (EMPs) play an important role here. In our study, there is negative correlation between age and lag time and positive correlation between CRP and etp and peak time. Increase in thrombosis tendency is an expected result. MPs increase in number and become more effective functionally with aging and this might contribute to this thrombosis tendency. Contrary to our study, Forest et al. showed that MPs decreased in elderly, although EMPs procoagulant activity persisted [13]. This might be a result of the decrease in circulating endothelium progenitor cells and decrease in endothelium differentiation.

In the presence of hypertension, endothelial dysfunction increases EMPs. Brodsky et al. showed that it caused impairment in vasorelaxation by decreasing endothelial NO production or bioavailability [28]. In a similar study, it was found that EMPs induce the expression of endothelial cyclooxygenase type 2, different adhesion molecules, release of cytokines, and impaired release of NO from vascular endothelial cells [29]. Burger et al. showed that endothelial expression of vascular cell adhesion molecule 1 (VCAM-1), platelet endothelial cell adhesion molecule (PECAM-1) increased endothelial excretion and contributed to endothelial inflammation [30]. Boulanger et al. [31] showed that MPs impaired endothelial NO transduction pathway in myocardial infarction and caused endothelial dysfunction. Martin et al. [32] showed similar findings in T cell originated MPs. High EMPs level may be considered as a biomarker of vascular damage [33].

Preston et al. [34] compared untreated hypertensive patients of stage 2 and 3 with healthy people. In stage 3 hypertensive patients, increased EMPs and platelet originated MPs (PMPs) was correlated with both systolic and diastolic blood pressure values. On the other hand, the change in stage 1 hypertension was not significant. As EMPs and PMPs increase coagulation, this might contribute to target organ damage of the hypertension. Cardoza et al. [35] found that angiotensin- 2 stimulation increased MPs secretion from mononuclear cells. They argued that angiotensin receptor 2 related increases in procoagulant MPS generation, is a new system that relates renin-angiotensin system to thrombosis.

The migration of phosphatidylserine (PS) to the cell surface not only facilitates the formation of clotting complexes, but also facilitates TF initiation of clotting. MPs support coagulation with extrinsic pathways (FVII/TF dependent and independent). The procoagulant activity of endothelial derived MPs (EDMPs), platelet derived MPs (PDMPs), monocyte derived MPs (MDMPs) is dependent on FVII/TF; our method of TGT works in compliance with this procoagulant activity.

MPs include bioactive phospholipids, various antigens that are characteristic of the cell to which they are source, their warning type and cytoplasmic components. The greatest advantage of the flow-cytometer is the double staining of MPs to determine the cellular source of MPs. Annexin V binding is used to confirm MPs phospholipid properties, but most EDMPs do not express this antigen. Antibodies against specific surface antigens (glycoproteins) expressed over source cells are used to identify the subtype of MPs (eg, anti-GPIb for the identification of PDMPs). Information is also obtained about the MPs dimensions by evaluating the forward light distribution of MPs with the flow-cytometer. MPs are also released from leukocytes, erythrocytes, endothelial cells, smooth muscle cells and cancer cells. Heterogeneity is an important feature of MPs. The same cells treated with different stimuli release the MPs carrying the different components. In contrast, different cell types treated with the same stimulus will also release MPs carrying different components [36–38].

In our study, finding no difference between the groups might be explained with that the patients were on treatment. It is shown that MPs are decreased in diabetic patients who take losartan or simvastatin [39]. Other studies also showed that angiotensin 2 receptor blockers and other antihypertensive drugs decreased MPs levels [40, 41]. The disappearance of the correlations between MPs parameters and age or CRP or other PLT indices in the patient group may be as a result of the anti-inflammatory or other effects of the drugs.

In patients with hypertension, mean PLT counts was found significantly higher in comparison with the controls; however, it was under the reference limit of 450 (× 10^9/L). This finding may be discussed in terms of hypertension development as well as the contribution of hypercoagulability to cardiovascular morbidity and mortality. Increased neutrophil and PLT counts were reported in a cohort study with elder adults aged 79–87 years [42] in another study, seasonal increase of mean platelet volume (MPV) and fibrinogen levels were recorded without any variation in PLT counts [43]. In our study, platelet indices; MPV, platelet distribution width (PDW) and platelet larger cell ratio (P-LCR) showed negative correlation with ETP and peak time. P-LCR is an indicator of circulating larger platelets (> 12 fL), which is presented as percentage. It has also been used to monitor platelet activity [44]. Also, increase in MPV and PDW shows thrombocyte activation. It is argued that it correlates with circulating PMPs and contributes to thrombosis. It is shown that circulating PMPs and MPs are increased acute ischemic stroke patients [45].

### Limitations of the study

We were not able to determine the MPs dimensions, so soluble factors and exosomes could influence the measured parameters.

## Conclusion

Our present findings can be taken into consideration in understanding the hemostasis via TGT parameters, in the elderly. Attention should be paid to contribution of MPs to thrombosis tendency seen with aging. Also, endothelial dysfunction, caused by HT, increases the number of circulating MPs. MPs might be considered as vascular inflammation and damage markers and used as follow up tools of medical treatment of vascular inflammation-related diseases.

## Data Availability

The data sets used and analyzed during the current study are available from the corresponding author on reasonable request.

## Abbreviations

CRP: C- reactive protein
EMPs: Endothelial derived MPs
EPCs: Endothelial progenitor cells
ETP: Endogenous thrombin potential
MPs: Microparticles
MPV: Mean platelet volume
PDW: Platelet distribution width
P-LCR: Platelet larger cell ratio
PLT: Platelet
PMPs: Platelet originated MPs
TGT: Thrombin generation test
TtPeak: Time to thrombogram peak
VCAM-1: Vascular cell adhesion molecule-1
